# Genome Sequence of the Thermophilic Biomass-Degrading Fungus *Malbranchea cinnamomea* FCH 10.5

**DOI:** 10.1128/genomeA.00779-17

**Published:** 2017-08-17

**Authors:** Zoraide Granchi, Sake van Pelt, Vu Nguyen Thanh, Lisbeth Olsson, Silvia Hüttner

**Affiliations:** a GenomeScan B.V., Leiden, The Netherlands; b Center for Industrial Microbiology, Food Industries Research Institute, Thanh Xuan, Hanoi, Vietnam; c Division of Industrial Biotechnology, Department of Biology and Biological Engineering, Chalmers University of Technology, Gothenburg, Sweden; d Wallenberg Wood Science Center, Chalmers University of Technology, Gothenburg, Sweden

## Abstract

We report here the annotated draft genome sequence of the thermophilic biomass-degrading fungus *Malbranchea cinnamomea* strain FCH 10.5, isolated from compost at a waste treatment plant in Vietnam. The genome sequence contains 24.96 Mb with an overall GC content of 49.79% and comprises 9,437 protein-coding genes.

## GENOME ANNOUNCEMENT

*Malbranchea cinnamomea* is a thermophilic fungus belonging to the order *Onygenales* (1) that is naturally found in composting soil and has the capability of degrading plant biomass. It is a promising source of thermostable enzymes, such as α-glucosidases, xylanases, α-amylases, and glucanases, that have industrial relevance as biocatalysts, particularly in biorefinery contexts (2–5). To be able to fully exploit the enzymatic system of *M. cinnamomea* and investigate its strategy of growth on various lignocellulosic materials, we sequenced the strain FCH 10.5, which was isolated from compost at the Cau Dien waste treatment factory in Hanoi, Vietnam. The fungus was cultivated at 50°C for 48 h in liquid medium with the following composition: 4 g liter^−1^ KH_2_PO_4_, 13.6 g liter^−1^ (NH_4_)_2_SO_4_, 0.8 g liter^−1^ CaCl_2_-2H_2_O, 0.6 g liter^−1^ MgSO_4_-7H_2_O, 6 g liter^−1^ Bacto peptone, 10 mg liter^−1^ FeSO_4_-7H_2_O, 3.2 mg liter^−1^ MnSO_4_-H_2_O, 2.8 mg liter^−1^ ZnSO_4_-7H_2_O, 4 mg liter^−1^ CoCl_2_−6H_2_O, 200 mL liter^−1^ Tween 80, and 20 g liter^−1^ glucose. DNA for sequencing was extracted with cetyltrimethylammonium bromide (CTAB) buffer (2% CTAB, 100 mM Tris HCl, pH 8.0, 20 mM EDTA, 1.4 M NaCl) and purified from the supernatant by a combination of phenol-chloroform extraction and isopropanol precipitation (6) and a DNeasy Plant minikit (Qiagen).

Genome sequencing was performed in GenomeScan facilities. The NEBNext Ultra DNA library prep kit for Illumina (catalog no. NEB E7370S/L) was used according to the manufacturer’s protocol for library preparation. Quality and yield after sample preparation were measured using Bioanalyzer (Agilent Technologies).

Clustering and DNA sequencing using the Illumina cBot and HiSeq 2500 systems were performed according to the manufacturer’s protocols using a concentration of 8.0 pM of DNA, standard Illumina primers, and HiSeq control software HCS version 2.2.58. Image analysis, base calling, and quality checking were performed with the Illumina data analysis pipeline RTA version 1.18.64 and Bcl2fastq version 1.8.4.

The 250-nt paired-end reads were trimmed for adapter sequences and filtered for sequence quality using our in-house tool FASTQFilter version 2.05. The short-read genome assembler based on De Bruijn graphs, Abyss version 1.3.7 (7), was used for assembly with an optimized *k*-mer length of 64. Scaffolds shorter than 500 bp were removed.

For gene finding, two approaches were used and combined to obtain the presented data. The HMM-based algorithm Glimmer version 3.02 (8) was trained using the genome of *Uncinocarpus reesii* (downloaded from JGI, http://genome.jgi.doe.gov). Furthermore, mapped mRNA-Seq reads were used by the CodingQuarry (9) software tool for an evidence-based method of gene finding. Combining the two methods, gene models for 9,437 genes were obtained. For annotation, coding sequences were translated into amino acid sequences, and a BLASTp search (version 2.2.28+) was performed on the SwissProt database with default parameters.

### Accession number(s).

The *M. cinnamomea* FCH 10.5 whole-genome sequence has been deposited in DDBJ/EMBL/GenBank under the assembly accession no. FQSS00000000. The version described in this paper is the second version, FQSS02000000.
